# Comparison of medication adherence between type 2 diabetes mellitus patients who pay for their medications and those who receive it free: a rural Asian experience

**DOI:** 10.1186/s41043-019-0161-9

**Published:** 2019-01-24

**Authors:** Devarajan Rathish, Ruvini Hemachandra, Thilini Premadasa, Sasini Ramanayake, Chathuri Rasangika, Ravi Roshiban, Channa Jayasumana

**Affiliations:** Department of Pharmacology, Faculty of Medicine and Allied Sciences, Rajarata University of Sri Lanka, Saliyapura, Sri Lanka

**Keywords:** Non-adherence, Community support, Diabetes mellitus, Rural sector, Sri Lanka, Universal-free

## Abstract

**Background:**

Treatment plans fail if patients have poor medication adherence. Our aim was to compare medication adherence, reasons for non-adherence, and satisfaction with community support among type 2 diabetes mellitus patients who pay for their medications and those who receive it free.

**Methods:**

A descriptive cross-sectional study was conducted at Anuradhapura, Sri Lanka, among patients who were on oral anti-diabetic drugs for at least 3 months. They were grouped into two: universal-free group and fee-paying group. Three different scales were used to score medication adherence, reasons for non-adherence, and satisfaction with community support. Fisher’s exact test was performed to determine if there was a significant difference between the two groups (*p* < 0.05) concerning medication adherence and satisfaction with community support.

**Results:**

The median (IQR) medication adherence scores for fee-paying group and universal-free group were 3 (2-3) and 3 (3-3), respectively; the median (IQR) scores for satisfaction with community support were 5 (2–6) and 4 (4–6), respectively. Both the adherence and the satisfaction failed to show a significant difference between the two groups. Forgetfulness, being away from home, complex drug regime, and willingness to avoid side effects were common reasons of non-adherence for both the groups.

**Conclusions:**

There was no significant difference in medication adherence between the universal-free group and fee-paying group, despite of having a significantly different income. The universal-free health service would be a probable reason.

**Electronic supplementary material:**

The online version of this article (10.1186/s41043-019-0161-9) contains supplementary material, which is available to authorized users.

## Background

The goal of prescribed medical therapy is to improve the patient’s disease condition. Despite efforts of healthcare professionals, achievement of this goal may be impeded if the patients are non-adherent to medical advice and treatment [1]. Adherence to long-term therapy is defined as “the extent to which a person’s behaviour – taking medication, following a diet, and/or executing lifestyle changes, corresponds with agreed recommendations from a health care provider” [2, 3]. The disadvantages of non-adherence are the waste of medication, disease progression, reduced functional abilities, poor quality of life, and increased use of medical resources [4]. There are several factors associated with non-adherence to medication. These are healthcare team/health system factors, socio-economic factors, therapy-related factors, illness-related factors, and the patient-related factors [5]. The socio-economic factors include poor socioeconomic status, high cost of transport and medication, unemployment, lack of social support, and long distance from hospitals [5].

Non-adherence to prescribed treatment is a leading problem among patients with non-communicable diseases (NCD) worldwide and medication adherence in developed countries was only 50% [3, 6]. In developing countries, adherence is much lower than developed countries due to the lack of resources and poor access to resources [3, 6]. NCD are slowly progressive chronic diseases and patients have to live with it. This might be a cause for poor medication adherence in NCD [7]. In addition, people with NCD are on concurrent use of medications due to the prevalence of multiple risk factors. NCD bring a large burden on human health worldwide. Currently, NCD cause more than 60% of all deaths. Roughly four out of five NCD deaths in 2008 occurred in low- and middle-income countries [8].

World health Organisation (WHO) projects that diabetes mellitus (DM) will be the seventh leading cause of death in 2030 [9]. According to the International Diabetes Federation (IDF), the global prevalence of diabetes mellitus among the age group 20–79 years was 8.8% in 2015. This may increase up to 10.4% in 2040 [10]. Seventy-five percent of the people with DM live in low- and middle-income countries. More than half (56%) of all DM patients were from Southeast Asian region or West Pacific region in 2015. According to a recent IDF data, the prevalence of DM among adults in Sri Lanka was 8.5%, and currently, 1 in 12 adults of Sri Lanka has DM [11].

In the management of DM, glycemic control plays a major role and this is influenced by the patient’s medication adherence [12, 13]. Therefore, it is essential to assess the medication adherence to achieve an effective DM management [14]. Most of the previous studies on adherence to anti-DM medication have shown low adherence pattern to both pharmacological and non-pharmacological therapies [15, 16]. Worldwide adherence rate for anti-DM medication fluctuates between 36 and 93% [17]. Several studies have shown that low income and low educational levels have been associated with higher rates of non-adherence to anti-DM agents [18–21]. Side effects such as gastrointestinal effects, hypoglycemia, and weight gain have led to poor adherence to anti-DM treatment [22–24]. In addition, patients on multiple, complex therapy had poor adherence compared to patients on monotherapy [22].

According to IDF Diabetes Atlas, the number of adults with DM in Sri Lanka will increase from 1,080,000 in 2011 to 1,467,000 by 2030 [25]. Poor economy and poor infrastructure at rural regions would result in poor accessibility to healthcare services and subsequent poor medication adherence [18–21]. Therefore, it is essential to undertake a study on medication adherence among type 2 DM patients of these regions. In addition, each community has its own culture and lifestyle that may affect adherence. A recent study with similar objectives was done in an urban area of Sri Lanka [26]. It had 35.8% adherence at a medical clinic of a tertiary care government hospital (universal- free) and 12.6% at private sector clinics (fee- levying). However, studies are scarce on medication adherence among DM patients of rural Sri Lanka and on comparison of adherence between patients who pay for their medications and those who receive it free. Thus, we hope to conduct a study to compare medication adherence among the above-mentioned two groups in Anuradhapura. In addition, the study focuses on finding the reasons given by patients for non-adherence and the satisfaction with community support they receive for their treatment.

## Methods

A descriptive cross-sectional study was conducted during the month of August 2017.

### Study setting

The study setting for the selection of patients who pay for their medication (fee-paying group) was the State Pharmaceutical Corporation (SPC), Anuradhapura, Sri Lanka. The prevalence of DM is 9.6% in North Central Province [27]. Anuradhapura is the largest district by surface area in the North Central Province and in Sri Lanka, where the population is of nearly 856,500 by 2012. The majority of its population (94.6%) belongs to the rural sector [28]. The mean monthly household income of Anuradhapura district is 35,460 Sri Lankan rupees which is low compared to the overall mean monthly household income of the country (45,878 Sri Lankan rupees) [29]. SPC’s national role is to serve Sri Lanka by providing safe, efficacious, and high-quality medicinal products at affordable prices while promoting the use of generic drugs compared to private pharmacies in the country [30]. The only outlet of SPC in Anuradhapura is situated within 500 m from Teaching Hospital Anuradhapura. The next outlet of SPC is either in Polonnaruwa, Kurunegala, or Jaffna districts which are 100, 115, 200 km away, respectively. In addition, major private DM clinics are within 500 m from the SPC, Anuradhapura. A recent study done at SPC, Anuradhapura, found metformin and gliclazide being two of the top 10 medications prescribed from both government and private institutes in Anuradhapura, Sri Lanka [31]. Certain anti-diabetic agents like Dipeptidyl peptidase-4 inhibitors are not available in the universal-free government hospitals but are found at SPC. Therefore, a large number of low-income and rural population visit the aforementioned SPC to obtain anti-diabetic drugs for a fee.

One pharmacy each from the universal-free (government-owned) primary, secondary, and tertiary healthcare institutes of Anuradhapura was chosen as study settings for the selection of patients who receive medications free of charge (universal-free group). Teaching Hospital Anuradhapura (THA) was one of them. THA is the only tertiary care hospital available for the entire North Central Province, which is owned by the state. This makes it the only choice for patients of Anuradhapura to seek specialized care for diabetes mellitus. The next setting was Base Hospital Thambuttegama (BHT), a secondary care institution. BHT is the only type-A base hospital of Anuradhapura; therefore, it is the highest graded among the secondary care institutions of Anuradhapura. The last setting was Divisional Hospital Kekirawa (DHK), a primary care institution, which was randomly selected among the four divisional hospitals of Anuradhapura district.

### Sample size

The minimum sample size was calculated as 50 using data from previous literature [26] and the formula: *n* = (*Z*_*α*/2_+*Z*_*β*_)^2^ × [*P*1(1-*P*1) + *P*2(1-*P*2)]/(*P*1-*P*2)^2^

where *Z*_*α*/2_ is the type I error = 1.96, *Z*_*β*_ is the power = 0.84, *P*1 is the adherence among the universal-free group = 35.8% [26], *P*2 is the adherence among the fee-paying group = 12.6% [26], and *n* is the sample size = 50.

Fifty patients each were recruited from SPC, THA, BHT, and DHK. The total number of patients recruited from the universal-free group was 150 (fee-paying group:universal-free group = 1:3). Male to female ratio was maintained at 1:1 in all institutions.

### Sampling method and selection criteria

All consecutive eligible males and females presented to each of the institutes were sampled separately until the minimum sample size was achieved for each gender (*n* = 25). By this, a sample size of 50 and a male to female ratio of 1:1 were maintained at each institute. Four separate working days of August 2017 were selected for data collection at SPC, THA, BHT, and DHK, respectively. The inclusion criteria were as follows: oral anti-diabetic drugs (obtained from the particular pharmacy) for the last 3 months or more, age equal or more than 18 years, and permanent residence of Anuradhapura district for at least 5 years. The exclusion criteria were as follows: having a history of or currently on self-use of insulin, GLP-1 agonist or amylin mimetics, pregnant mothers, and cognitive impairment.

### Instruments

A self-administered questionnaire was used to gather demographic data. The gathered information was entered in a Microsoft Excel sheet (Additional file 1). Morisky, Green, and Levine adherence scale (permission was obtained from the corresponding author via an e-mail) was used to assess medication adherence among study participants [21]. The four questions of the scale were administered by trained MBBS qualified doctors. The scale ranged from 0 to 4 with 0 being low adherence and 4 being high adherence. If an item was marked as “yes,” it was scored as 0, and if it was “no,” it was scored as 1. The categorical scoring for adherence was as follows: 2–4 for adherence (moderate and high) and 0–1 for non-adherence (low).

Relevant sections of the Culig adherence scale (permission was obtained from the corresponding author via an e-mail) were used to find the reasons for non-adherence among study participants and to find the satisfaction of patients with community support received for their treatment [32]. These were self-administered with instructions and help provided by trained MBBS qualified doctors. The scoring ranged from 0 to 3. For causes of non-adherence, the scoring was as follows: 0 - never; 1- very rare (occurring 1–2 times per year); 2- sometimes (occurring 3–5 times per year); and 3- often (occurring > 5 times per year). For satisfaction with community support, the scoring was as follows: 0- I am very unsatisfied; 1- I am mostly unsatisfied; 2- I am mostly satisfied; and 3- I am very satisfied. The categorical scoring for satisfaction with community support was as follows: 0–1 for unsatisfied and 2–3 for satisfied.

### Data collection, data analysis, and description of data

Study description, obtaining informed written consent, and data collection were done by trained MBBS qualified doctors under the supervision of the principal investigator (DR). Data was analyzed using Microsoft Excel. Data cleaning and verification of random samples of the digital data against the original data were done to assure data quality. Medians with interquartile ranges (IQR) were presented as the data were not normally distributed. Fisher’s exact test was used to find a significance (*p* < 0.05) between the two groups for the following: proportions of participants having high adherence (scores 2–4 of Morisky, Green, and Levine adherence scale) and proportions of participants being satisfied with the community support (scores 2–3 of Culig adherence scale). Reasons for non-adherence were ranked according to the average scores achieved.

## Results

### Demographic features

Most were educated up to grade 9–11 among the fee-paying group (52%) and the universal-free group (40%). Most were married (by registration) among participants at SPC (82%), THA (90%), BHT (86%), and DHK (68%). Most of the participants at SPC (56%), THA (52%), BHT (70%), and DHK (86%) fell under the category “occupations none, unidentifiable.”

Percentage living alone was 8% and 6% for the fee-paying group and the universal-free group, respectively. The median (IQR) number of drugs used by participants per day for the last 1 month was 3 (3–5), 4 (3–7), 3 (3–5), and 3 (2–4) for SPC, THA, BHT, and DHK, respectively. Monthly salary was significantly higher in the fee-paying group compared to the universal-free group (*p* < 0.01). Demographic features and co-morbidities of the study participants are summarized in Table 1.

**Table 1:** Characteristics of the study participants— medication adherence study, Anuradhapura 2017

Items	Fee-paying group—SPC (*n* = 50)	THA (*n* = 50)	BHT (*n* = 50)	DHK (*n* = 50)	Universal-free group (*n* = 150)	*p* value*
Demographic data						
Median age (IQR)	59 (52–65)	59 (54–65)	60 (49–67)	60 (54–67)	60 (52–66)	0.73^#^
Years residing at Anuradhapura (IQR)	47 (35–58)	57 (47–63)	40 (28–57)	56 (40–62)	52 (36–61)	0.17^#^
Median monthly salary (rupees) (IQR)	30,000 (23,684–40,250)	15,000 (9750–25,000)	8000 (3000–15,000)	10,000 (2000–25,000)	15,000 (5000–25,000)	< 0.01^#^
Data related to co-morbidities						
Median duration of T2DM (months) (IQR)	66 (33–135)	60 (23–147)	72 (33–120)	72 (36–135)	60 (36–120)	0.56^#^
Percentage with any level of visual Impairment (%)	54	50	58	68	59	0.56^$^
Percentage with hyperlipidemia (%)	42	58	44	36	46	0.62^$^
Percentage with hypertension (%)	42	56	52	58	55	0.10^$^

*SPC* State Pharmaceutical Corporation, *THA* Teaching Hospital Anuradhapura, *BHT* Base Hospital Thambuttegama, *DHK* Divisional Hospital Kekirawa, *T2DM* type 2 diabetes mellitus, *IQR* interquartile range

*p value was calculated for the fee-paying group against the universal-free group (THA, BHT, DHK combined)

^#^Mann-Whitney *U* test was performed

^$^Chi-square test was performed

### Medication adherence

**Fig. 1:**
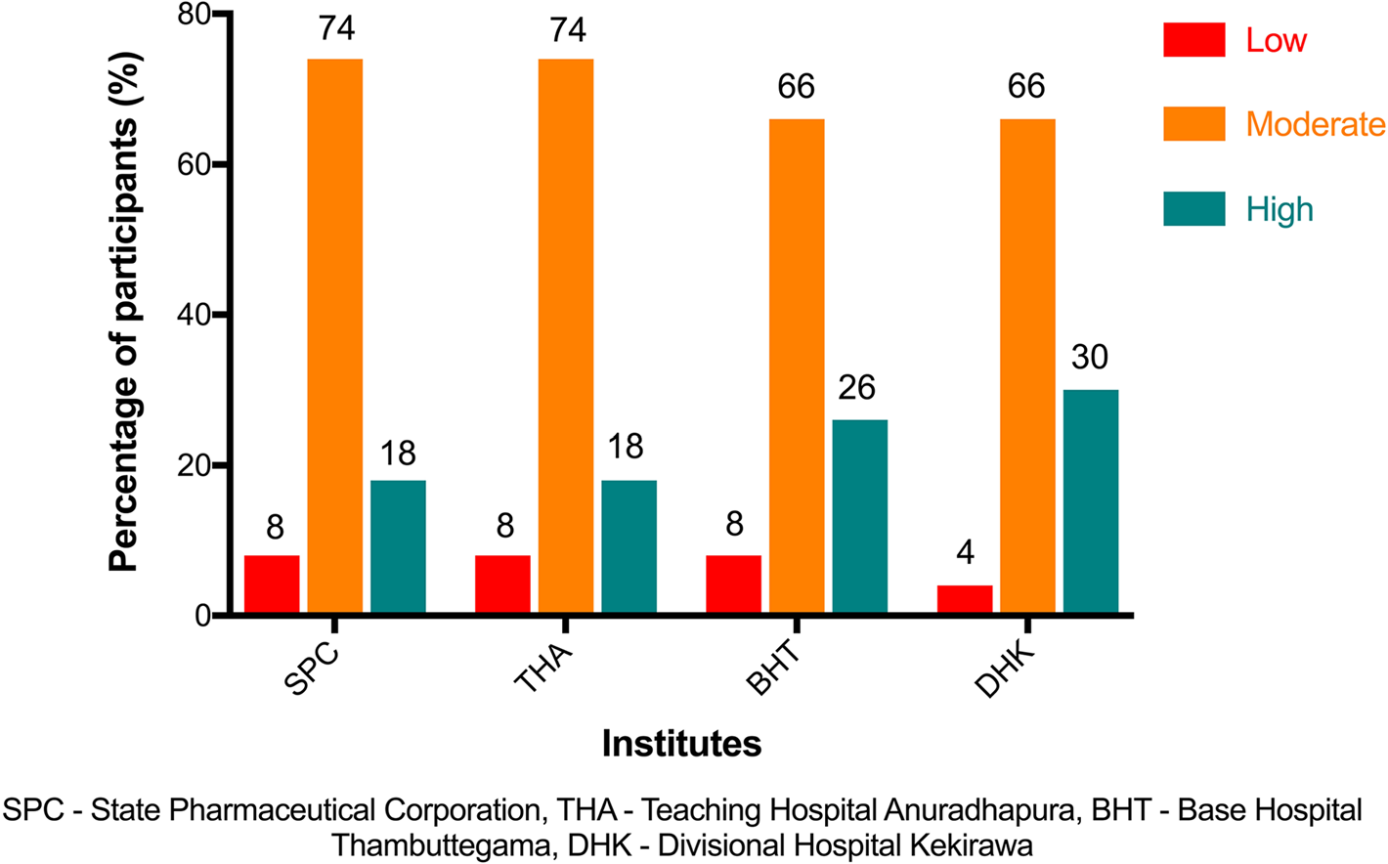
Percentage of participants with low, moderate, and high medication adherence by institute—medication adherence study, Anuradhapura 2017

Overall, the mean medication adherence score for the universal-free group (THA, BHT, and DHK) was 3 (3-3) in comparison to 3 (2-3) of the fee-paying group. The breakdown of medication adherence scores concerning each institution is shown in Table 2. The fee-levying SPC had 8% (*n* = 50) for non-adherence (low); it was 92% for adherence (moderate and high). Overall, the universal-free group had 7% (*n* = 150) for non-adherence (low); it was 93% for adherence (moderate and high). There was no significant difference between the two groups for the proportions of participants having high adherence (*p* = 0.96). Figure 1 shows the breakdown of adherence for each pharmacy.

**Table 2:** Median (IQR) scores for the four items of Morisky, Green, and Levine adherence scale

Items	SPC (fee-paying group) (*n* = 50)	THA (*n* = 50)	BHT (*n* = 50)	DHK (*n* = 50)	Universal-free group (*n* = 150)
Do you ever forget to take your medicine?	0 (0–0)	0 (0–1)	0 (0–1)	0 (0–1)	0 (0–1)
Are you careless at times about taking your medicine?	1 (1–1)	1 (0–1)	1 (0–1)	1 (1–1)	1 (1–1)
When you feel better, do you sometimes stop taking your medicine?	1 (1–1)	1 (1–1)	1 (1–1)	1 (1–1)	1 (1–1)
Sometimes if you feel worse when you take the medicine, do you stop taking it?	1 (1–1)	1 (1–1)	1 (1–1)	1 (1–1)	1 (1–1)
Median (IQR) score for medication adherence (Overall)	3 (2–3)	3 (2–3)	3 (3–4)	3 (3–4)	3 (3–3)

*SPC* State Pharmaceutical Corporation, *THA* Teaching Hospital Anuradhapura, *BHT* Base Hospital Thambuttegama, *DHK* Divisional Hospital Kekirawa, *IQR* interquartile range

### Reasons given by patients for non-adherence

Forgetfulness (mean score = 1.3), being away from home (1.0), complex drug regime (0.5), willingness to avoid side effects (0.4), and too expensive medications (0.2) were the top five reasons for non-adherence at universal-free (THA, BHT, DHK combined) group. The top five reasons for non-adherence in each of the pharmacies are shown in Table 3.

**Table 3:** Top five reasons for non-adherence—medication adherence study, Anuradhapura 2017

No.	SPC (*n* = 50) Reason and mean (SD)		THA (*n* = 50) Reason and mean (SD)		BHT (*n* = 50) Reason and mean (SD)		DHK (*n* = 50) Reason and mean (SD)	
01	Forgetfulness	1.4 (1.3)	Being away from home	1.4 (1.2)	Forgetfulness	1.0 (1.1)	Forgetfulness	1.5 (1.3)
02	Being away from home	1.2 (1.2)	Forgetfulness	1.3 (1.2)	Being away from home	0.8 (1.1)	Being away from home	0.8 (1.2)
03	Running out of medications	0.9 (1.2)	Willingness to avoid side effects	0.6 (1.2)	Complex drug regime	0.6 (1.1)	Complex drug regime	0.3 (0.8)
04	Complex drug regime	0.4 (1.0)	Complex drug regime	0.4 (0.9)	Willingness to avoid side effects	0.4 (0.9)	Felt depressed or broken	0.3 (0.7)
05	Willingness to avoid side effects	0.4 (0.9)	Felt depressed or broken	0.2 (0.7)	Felt well	0.2 (0.7)	Too expensive medications	0.3 (0.8)
05			Felt well	0.2 (0.8)	Too expensive medications	0.2 (0.8)		

*THA* Teaching Hospital Anuradhapura, *BHT* Base Hospital Thambuttegama, *DHK* Divisional Hospital Kekirawa, *SD* standard deviation

### Satisfaction with community support

**Fig. 2:**
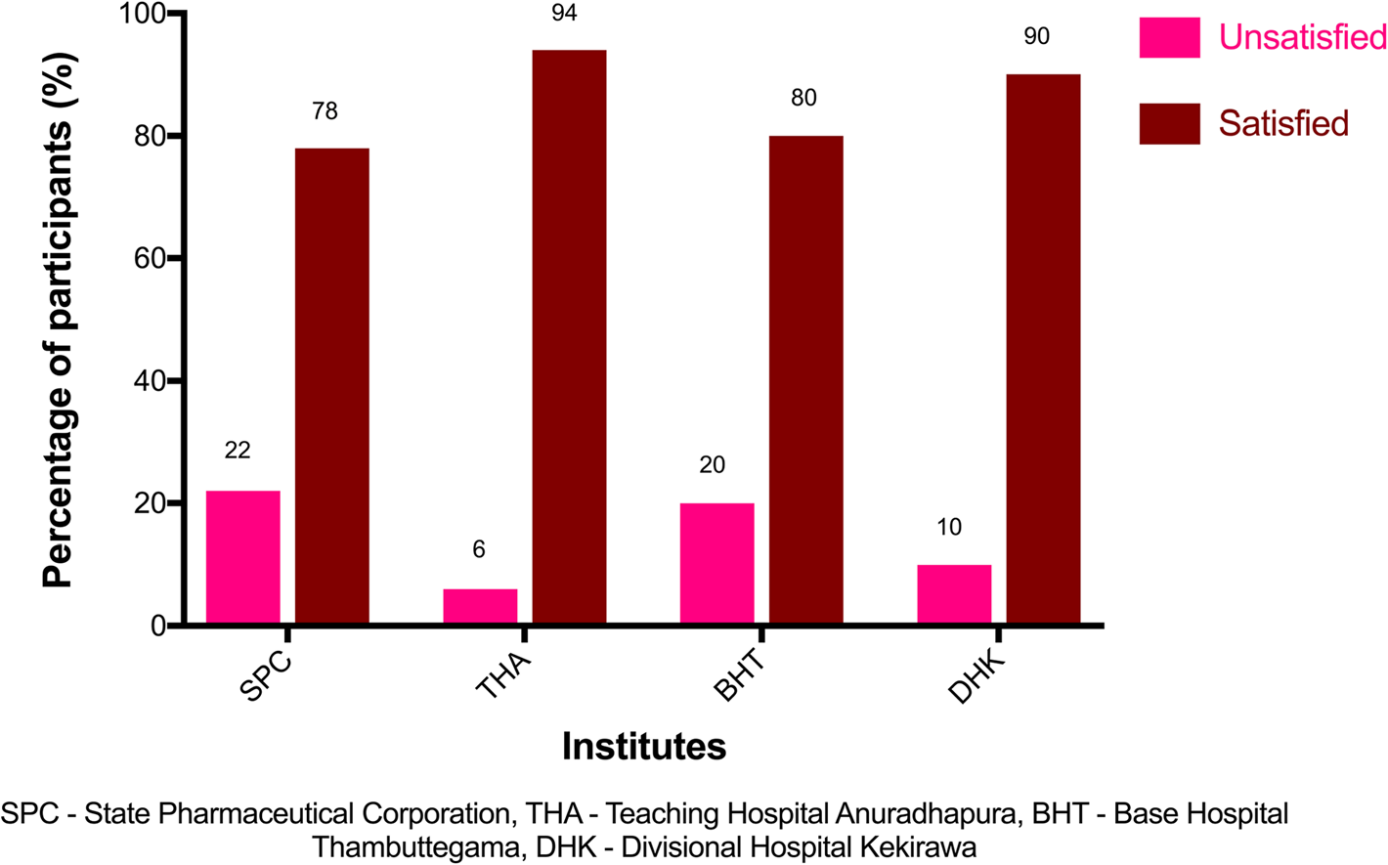
Percentage of participants satisfied with community support for each institute—medication adherence study, Anuradhapura 2017

Overall, the median (IQR) score for satisfaction with community support for universal-free group (THA, BHT, and DHK) was 4 (4–6) in comparison to 5 (2–6) of the fee-paying group. The breakdown of scores for satisfaction with community support for the two items of the Culig adherence scale is shown in Table 4. The fee-levying SPC had 22% (*n* = 50) of unsatisfied participants for community support. Overall, the universal-free group had 12% (*n* = 150) of unsatisfied participants for community support. There was no significant difference between the two groups for the proportions of satisfied participants (*p* = 0.14). Figure 2 shows the breakdown of satisfaction with community support for each pharmacy.

**Table 4:** Median (IQR) scores for the 2 items of Culig adherence scale—medication adherence study, Anuradhapura 2017

Items	SPC (fee-paying group) (*n* = 50)	THA (*n* = 50)	BHT (*n* = 50)	DHK (*n* = 50)	Universal-free group (*n* = 150)
Are you satisfied with the support of your family and friends?	2 (1–3)	2 (2–3)	3 (1–3)	2 (2–3)	2 (2–3)
Do your family and friends remind you to take medication on time?	2 (1–3)	2 (2–3)	3 (1–3)	2 (2–3)	2 (2–3)
Median (IQR) scores for satisfaction with community support (Overall)	5 (2–6)	4 (4–6)	6 (2–6)	5 (4–6)	4 (4–6)

*SPC* State Pharmaceutical Corporation, *THA* Teaching Hospital Anuradhapura, *BHT* Base Hospital Thambuttegama, *DHK* Divisional Hospital Kekirawa, *IQR* interquartile range

## Discussion

Medication adherence and satisfaction with community support failed to show a significant difference between the fee-paying group and the universal-free group, despite a significant difference in the monthly income of the two groups.

According to a previous study, there is a higher risk of hospitalization of type 2 DM patients during the following year if they fail to obtain at least 80% of their oral anti-diabetic drugs for 1 year [33]. In-addition, previous findings on adherence to anti-DM drugs show low adherence [15, 16]. However, the percentage for moderate to high adherence among the DM patients was more than 90% (*n* = 50) at each of the pharmacies of this study. Sri Lankan data using a similar scale was scarce. Kavitha et al. had found adherence to be 70% among diabetic patients of Hassan, India, using the eight-item modified Morisky adherence scale [34]. A study from Uganda produced 83.3% adherence to anti-diabetic medications [35]. However, Sontakke et al. report 74% low adherence from Nagpur, India [36]. As mentioned earlier, the worldwide adherence rate for anti-DM medication varies between 36 and 93% [17]. The present study population is towards the upper limit.

The monthly salary of the fee-paying group was significantly higher compared to that of the universal-free group. More affluent have chosen the fee-levying pharmacy however, there were no significant differences in the medication adherence of the two groups. In contrary, the previous study with similar objectives conducted in an urban area of Sri Lanka had 35.8% adherence at a universal-free clinic compared to 12.6% at fee-levying private sector clinics [26]. Unfortunately, the publication had no results on a significance test. Although previous studies have shown that poor economy and poor accessibility to healthcare services are associated with higher rates of medication non-adherence [5], our study in a rural region had produced contradicting evidence. This highlights the possible positive impact of the universal-free healthcare service, especially among the rural dwellers. Nevertheless, there was a notable difference among the top five reasons for non-adherence between the two groups. “Running out of medications,” which was among the top five for the fee-paying group, was replaced by “too expensive medications” for the universal-free group. Expense is still a concern among patients who receive medications at universal-free pharmacies. A recent study revealed inadequacies in the availability of essential medicines at universal-free healthcare institutions of Anuradhapura [37]. Therefore, there might be an instance when the patient is expected to purchase part of the prescription from a fee-levying pharmacy. Another possible expense involved would be the traveling cost to visit these institutes from their respective villages. Inadequacies in public transport would have made patients to use private transport (for example a three-wheeler) which is much expensive. Interestingly, non-refill of prescriptions due to relatively high cost of medication has topped the list of practical barriers in a study done in Nigeria [38]. High cost has been found as a reason for non-adherence by other studies too [39, 40]. Other reasons, such as forgetfulness [34, 36, 38, 39, 41], being away from home [38, 39], complex drug regime [38, 41, 42], and willingness to avoid side effects [38, 39, 41, 42], were similar to previous literature.

Prior studies have stressed the importance of community support in improving adherence to medication [43, 44]. Literature points out at a promising relationship between community support and diabetes management [45]. Most of the participants of the present study were satisfied with the community support they received. Proportion satisfied with community support was higher among the universal-free group (88%) compared to the fee-paying group (78%). However, there was no statistically significant difference between the two groups.

The findings of this study are unique, as it has compared data on medication adherence between fee-paying and universal-free groups of DM patients of a rural region. However, it had its own limitations. Recruiting of participants from private sector pharmacies would have been ideal for further comparison. However, high cost of medications, lack of waiting area, and relatively poor attendance at the private pharmacies of Anuradhapura made us to choose the fee-levying state pharmacy at SPC for comparison. Medication adherence is influenced by several factors, which lead to multiple confounders. Exclusion of all these confounders would be methodologically challenging. In addition, the inability of the patients to identify individual drugs prevented us from assessing adherence against each drug type.

## Conclusion

Regardless of whether the medication was obtained free or for a fee and regardless of a significant difference in the monthly income, the medication adherence showed no significant difference. The finding could probably be credited to the universal-free healthcare system. The reasons for non-adherence have highlighted areas where there is a need for improvement in medication adherence among patients with chronic diseases. Further studies are essential to find probable interventions.

## Additional file

Additional file 1: Medication adherence among patients with type 2 diabetes mellitus of Anuradhapura, Sri Lanka, 2017. This provides the results of the entire study with data for each institution. (XLS 153 kb)

## Abbreviations

BHT: Base Hospital Thambuttegama
DHK: Divisional Hospital Kekirawa
DM: Diabetes mellitus
IDF: International Diabetes Federation
IQR: Interquartile range
MBBS: Bachelor of Medicine and Bachelor of Surgery
NCD: Non-communicable diseases
SL: Sri Lanka
SPC: State Pharmaceutical Corporation
THA: Teaching Hospital Anuradhapura
USD: United States dollars
WHO: World Health Organization
